# Glutathione Metabolism of the Brain—The Role of Astrocytes

**DOI:** 10.1111/jnc.70073

**Published:** 2025-05-02

**Authors:** Ralf Dringen, Christian Arend

**Affiliations:** ^1^ Center for Biomolecular Interactions Bremen, Faculty 2 (Biology/Chemistry) University of Bremen Bremen Germany; ^2^ Center for Environmental Research and Sustainable Technologies University of Bremen Bremen Germany

**Keywords:** astrocytes, cysteine, GSH, metabolic coupling, Mrps, oxidative stress

## Abstract

Astrocytes have essential functions in the brain as partners of neurons in many metabolic and homeostatic processes. The metabolism of the tripeptide GSH (γ‐L‐glutamyl‐L‐cysteinyl‐glycine) is an important example of a metabolic interaction between astrocytes and neurons. GSH is present in brain cells in millimolar concentrations and has essential functions as an antioxidant and as a substrate for detoxification reactions. A high GSH content protects astrocytes against oxidative stress and toxins and is therefore beneficial for the astrocytic self‐defense that helps to maintain the essential functions of astrocytes in the brain and will enable astrocytes to eliminate potential toxins before they may reach other brain cells. In addition, astrocytes provide neurons with the amino acids required for GSH synthesis in a process that involves the export of GSH from astrocytes by the multidrug resistance protein 1, the extracellular processing of GSH via the astrocytic γ‐glutamyl transpeptidase to generate the dipeptide cysteinyl‐glycine, and the extracellular cleavage of this dipeptide by the neuronal ectopeptidase aminopeptidase N. As GSH export from astrocytes strongly depends on the cytosolic GSH concentration, a high astrocytic GSH content will also facilitate GSH release and thereby the supply of GSH precursors to neighboring neurons. In this article, we will give an overview of the current knowledge on the GSH metabolism of astrocytes, address how a high astrocytic GSH content can help to maintain brain functions, and discuss open questions and future perspectives of research on the functions of astrocytes in the GSH metabolism of the healthy and diseased brain.

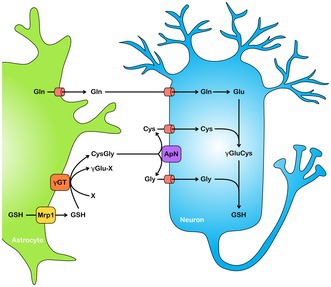

## Graphical abstract

Astrocytes supply neurons with the substrates for the synthesis of GSH (γGluCysGly). Astrocytes provide neurons with precursors for the three amino acids that are needed for GSH synthesis. The supply pathway involves the export of GSH from astrocytes via the multidrug‐resistance protein 1 (Mrp1), extracellular processing of GSH by astrocytic γ‐glutamyl transpeptidase (γGT) to the dipeptide cysteinyl‐glycine (CysGly) and hydrolysis of this dipeptide by neuronal aminopeptidase N (ApN) to cysteine (Cys) and glycine (Gly). Astrocyte‐derived glutamine (Gln) serves as a precursor for the glutamate (Glu) needed by neurons for GSH synthesis.

**Abbreviations**
ABC: ATP‐binding cassette
ApN: aminopeptidase N
BSO: buthionine sulfoximine
CysGly: cysteinyl‐glycine
EAATs: excitatory amino acid transporters
GCL: glutamate‐cysteine ligase
GCLm: modifier subunit of GCL
GlyT: glycine transporters
GPx: glutathione peroxidase(s)
GR: glutathione disulfide reductase
GS‐B: glutathione‐bimane adduct
GSH: glutathione
GSSG: glutathione disulfide
GST: glutathione S‐transferase
LDH: lactate dehydrogenase
Mrp: multidrug resistance protein(s)
MRS: magnetic resonance spectroscopy
MTT: 3‐(4,5‐dimethyl‐2‐thiazolyl)‐2,5‐diphenyl‐2H‐tetrazolium bromide
NAC: N‐acetyl cysteine
PPP: pentose‐phosphate pathway
PrP: prion protein
ROS: reactive oxygen species
γGluCys: γ‐glutamyl‐cysteine
γGT: γ‐glutamyl transpeptidase

## 1 Introduction

Astrocytes are important partners of neurons (Ciani et al. 2024; Zhang, Wang, et al. 2024) and have a plethora of important functions in the brain. For example, astrocytes substantially contribute to the ion and pH homeostasis in the brain (Theparambil et al. 2024; Untiet and Verkhratsky 2024), to the energy metabolism of the brain (Barros et al. 2024; Bonvento and Bolaños 2021; Chen et al. 2023) as well as to the detoxification of reactive oxygen species and toxins (Almeida et al. 2023; Dringen et al. 2015). In addition, astrocytes in the brain contribute to neurotransmitter homeostasis and neurotransmission (Andersen and Schousboe 2023; Cuellar‐Santoyo et al. 2023; Oliveira and Araque 2022) as well as to memory formation and maintenance (Bohmbach et al. 2023; Escalada et al. 2024). Furthermore, astrocytes are involved in the formation, maintenance, and function of the blood–brain barrier (Schiera et al. 2024). Astrocytes account for the majority of glial cells in the brain (Zhou et al. 2019) and represent 20% to 40% of all brain cells (Herculano‐Houzel 2014). In most brain areas, astrocytes fill 10% –15% of the total brain volume (Dienel and Rothman 2020). With their endfeet, astrocytes almost completely cover the brain capillaries (Cao et al. 2024; Mathiisen et al. 2010) and thus, astrocytes are the first parenchymal cells of the brain that encounter substances and drugs that enter the brain from the blood.

Glutathione (GSH, γ‐L‐glutamyl‐L‐cysteinyl‐glycine) is a tripeptide which has important functions in the antioxidative defense and in the detoxification of endogenous and exogenous compounds of almost all cell types (Deponte 2013; Georgiou‐Siafis and Tsiftsoglou 2023; Lapenna 2023). GSH can directly react chemically with radicals and serves as an electron donor for the reduction of peroxides by glutathione peroxidases (GPx). By these reactions, GSH is oxidized to glutathione disulfide (GSSG). Furthermore, GSH is a substrate of glutathione‐S‐transferases (GSTs) which detoxify endogenous compounds and xenobiotics by conjugation to GSH and thereby enable the cells to remove such compounds by active export of these conjugates (Dringen 2000). In the brain, millimolar concentrations of GSH are present within the cells (Dringen 2000), while extracellular GSH concentrations in the brain are orders of magnitude lower (Hilgier et al. 2010; Orwar et al. 1994).

**FIGURE 1:**
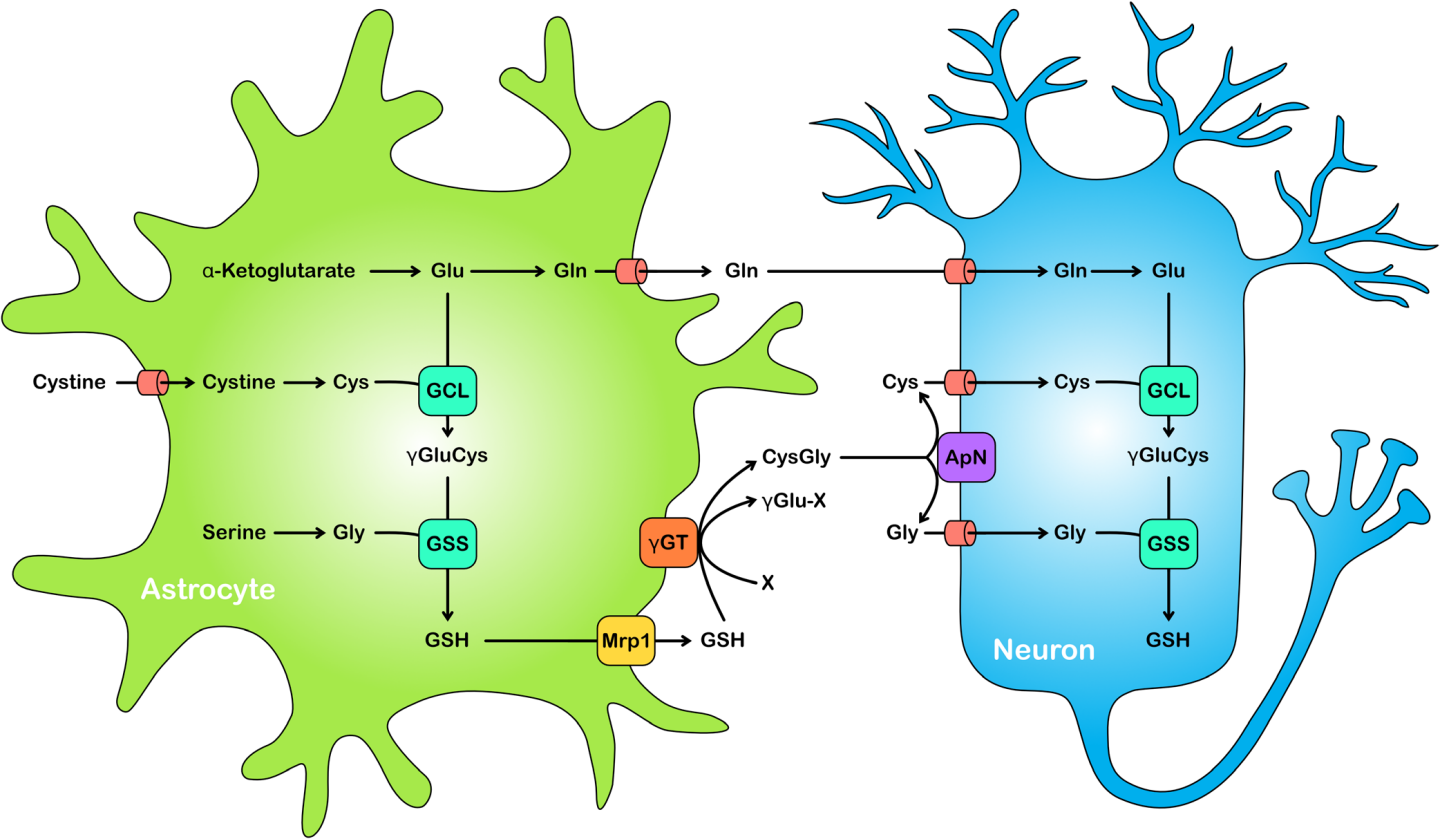
GSH synthesis in brain cells: Astrocytic supply of substrates for neuronal GSH synthesis. GSH is synthesized in astrocytes and neurons from the substrates glutamate (Glu), cysteine (Cys) and glycine (Gly) by the consecutive reactions of the two ATP‐consuming enzymes glutamate cysteine ligase (GCL) and GSH synthetase (GSS). The γGluCys produced from glutamate and cysteine by GCL is combined with glycine by GSS to form GSH (γGluCysGly). In astrocytes, glutamate and glycine are synthesized from α‐ketoglutarate and serine, respectively, while the cysteine needed for GSH synthesis is mainly derived from extracellular cystine (cysteine disulfide) that is taken up via the cystine‐glutamate antiporter X_C_^−^. GSH synthesis in neurons depends on the supply of the amino acid substrates from astrocytes, as they provide precursors for all three amino acids that are needed for neuronal GSH synthesis. The GSH exported from astrocytes via Mrp1 or other processes is processed by the astroglial ectoenzyme γ‐glutamyl transpeptidase (γGT). By transfer of the γ‐glutamyl moiety of GSH to an acceptor X, the dipeptide CysGly is liberated and subsequently hydrolyzed by the neuronal ectoenzyme aminopeptidase N (ApN). The generated amino acids cysteine and glycine are taken up by neurons and can be used as substrates for GSH synthesis. Additionally, astroglial glutamine synthetase amidates glutamate to glutamine (Gln), which is released and taken up by neurons as a precursor of the glutamate that is used as a neurotransmitter or for GSH synthesis.

Due to the strategically important location of astrocytes at the blood–brain barrier, a high GSH content empowers astrocytes to detoxify radicals, peroxides, and reactive compounds that may enter the brain from the periphery. However, this feature of astrocytes to modify and thereby inactivate xenobiotics has also to be considered in the context of the application of therapeutic drugs for the treatment of neurological disorders, as GSH‐dependent removal of such compounds in astrocytes may lower the therapeutic benefit of a given treatment. On the other hand, a high GSH content will help astrocytes to maintain their functions to protect the brain against oxidative stress and allows astrocytes to provide GSH precursors to neighboring neurons (Dringen et al. 2015; Dringen and Hirrlinger 2003; Dwivedi et al. 2020; Pérez‐Sala and Pajares 2023). Cysteine is the limiting substrate for GSH synthesis in neurons (Dringen, Pfeiffer, et al. 1999; Kranich et al. 1996). Astrocytes provide neurons with the required cysteine in a process that involves export of GSH from astrocytes, the extracellular processing of GSH via the astrocytic γ‐glutamyl transpeptidase (γGT) to generate the dipeptide cysteinyl‐glycine (CysGly) and the extracellular cleavage of this dipeptide by the neuronal ectopeptidase aminopeptidase N (Figure 1).

As astrocytes are considered to contain substantially higher GSH contents than neurons (Asanuma and Miyazaki 2021; Dringen et al. 2015; McBean 2017; Sedlak et al. 2019; Vázquez‐Meza et al. 2023), it can be concluded that a substantial part of the total GSH determined in brain tissue represents astrocytic GSH. As a consequence of the high abundance of astrocytes in the brain and of the well‐known pivotal role of astrocytes in brain GSH metabolism, it can be concluded that the reported loss in GSH in given brain areas in some human diseases (Detcheverry et al. 2024; Iskusnykh et al. 2022) is likely to be at least in part the consequence of a loss of astrocytic GSH and/or of alterations in the GSH metabolism of astrocytes.

The knowledge on the GSH metabolism of the brain has frequently been reviewed and updated (Aoyama 2021; Dringen 2000; Dwivedi et al. 2020; Pérez‐Sala and Pajares 2023; Rae and Williams 2017; Shaw 1998). In our article, we are going to focus mainly on the functions of astrocytes in the GSH metabolism of the brain and will discuss why a high GSH concentration in astrocytes can be beneficial for the brain. In addition, we will discuss open questions and future directions of research on the functions of astrocytes in the GSH metabolism of the brain.

## 2 GSH Synthesis in Astrocytes

GSH is synthesized from the amino acid substrates glutamate, cysteine, and glycine by the consecutive reactions of two ATP‐consuming cytosolic enzymes (Figure 1). The first of these two enzymes, the glutamate cysteine ligase (GCL) generates from glutamate and cysteine the dipeptide γ‐glutamyl‐cysteine (γGluCys). This enzyme catalyzes the rate‐limiting step in GSH synthesis and contains a catalytic and a modifier (GCLm) subunit. The GCL product γGluCys is subsequently connected by GSH synthase with glycine to generate GSH. The level of GSH in cells is regulated by feedback inhibition by GSH of GCL, the first enzyme in GSH synthesis (Franklin et al. 2009).

In astrocytes, the transcription factor Nrf2 plays an important role in the regulation of the synthesis of the enzymes responsible for GSH synthesis (Baxter and Hardingham 2016; He and Hewett 2025; Liddell 2017; Vargas and Johnson 2009). Accordingly, a number of Nrf2 activators, but also many other treatments, have been reported to increase the GSH content of cultured astrocytes (Dringen et al. 2015). In addition to the activity of the two enzymes involved in GSH synthesis, the availability of the amino acid substrates for GSH synthesis determines the rate of GSH synthesis and the GSH concentration in cultured rat astrocytes (Dringen and Hamprecht 1998; Kranich et al. 1998).

Cultured rat astrocytes have the potential to synthesize the three amino acids required for GSH synthesis from intracellular metabolites, but can also use extracellular amino acids and dipeptides as precursors of the three substrates of GSH synthesis (Dringen and Hamprecht 1996, 1998). Extracellular glutamate is efficiently taken up by the sodium‐dependent excitatory amino acid transporters (EAAT) 1 and 2 (Todd and Hardingham 2020), while glycine uptake is mediated by the sodium‐dependent transporters GlyT1 and GlyT2 (Marques et al. 2020). However, cultured rat astrocytes can also synthesize the glutamate needed for GSH synthesis from α‐ketoglutarate, glutamine, or proline and can generate glycine from serine (Dringen and Hamprecht 1996, 1998). Extracellular cysteine can also directly be taken up by cultured astrocytes and used for GSH synthesis, but its disulfide cystine, that is transported into rat astrocytes mainly by the cystine‐glutamate antiporter X_C_^−^, serves as the preferred extracellular substrate for the cytosolic cysteine needed for astrocytic GSH synthesis (Kranich et al. 1998, 1996; McBean 2017).

The cytosolic GSH concentration of cultured astrocytes was calculated to be 8 mM (Dringen and Hamprecht 1998). Part of the total GSH content of astrocytes is present in mitochondria (Huang and Philbert 1996; Yin et al. 2018). As GSH synthesis takes place exclusively in the cytosol, GSH has to be transported into mitochondria. In cultured rat astrocytes, both the dicarboxylate carrier and the 2‐oxoglutarate carrier of the inner mitochondrial membrane contribute to the mitochondrial uptake of GSH (Wilkins et al. 2013).

## 3 Modulation of the GSH Content of Astrocytes

A large variety of compounds and treatments has been reported to either increase or lower the GSH content of astrocytes, at least in culture (Dringen et al. 2015). Application of preferred extracellular substrates for GSH synthesis (Dringen and Hamprecht 1998; Kranich et al. 1998), a stimulation of the expression of GCL (Gegg et al. 2003; Lavoie et al. 2009) and a lowering of GSH consumption by inhibiting the continuous GSH export (Minich et al. 2006) have been shown to increase GSH contents in cultured murine astrocytes.

The loss in astrocytic GSH content by a given treatment can be caused by four main mechanisms: (1) impaired GSH synthesis due to lack of substrates (Dringen and Hamprecht 1996) or impaired activity of GSH synthesizing enzymes (Giordano et al. 2008; He and Hewett 2025; Lavoie et al. 2009), (2) conjugation of GSH to electrophiles (Ehrke et al. 2015; Schmidt and Dringen 2009, 2010), (3) accelerated export of GSH due to activation of the transporters mediating GSH export (Arend et al. 2013; Tulpule and Dringen 2011), and (4) the oxidation of GSH to GSSG which is subsequently exported from the cells (Hirrlinger et al. 2001; Minich et al. 2006).

Treatments and compounds that lower the GSH content of cultured murine astrocytes have previously been summarized (Dringen et al. 2015). More recently, GSH loss in cultured rat astrocytes was reported as a consequence of a stimulated Mrp1‐mediated GSH export in the presence of ritonavir (Arend et al. 2024) or octyl itaconate (Watermann et al. 2025), as well as by intracellular conjugation of GSH to electrophiles such as monochlorobimane (Arend et al. 2024) or dimethyl itaconate (Watermann et al. 2025). In addition, oxidation of intracellular GSH to GSSG and subsequent export of GSSG was recently reported for treatments of cultured rat astrocytes with menadione (Arend et al. 2024; Raabe et al. 2019; Steinmeier and Dringen 2019) and β‐lapachone (Steinmeier et al. 2020; Watermann et al. 2023).

**FIGURE 2:**
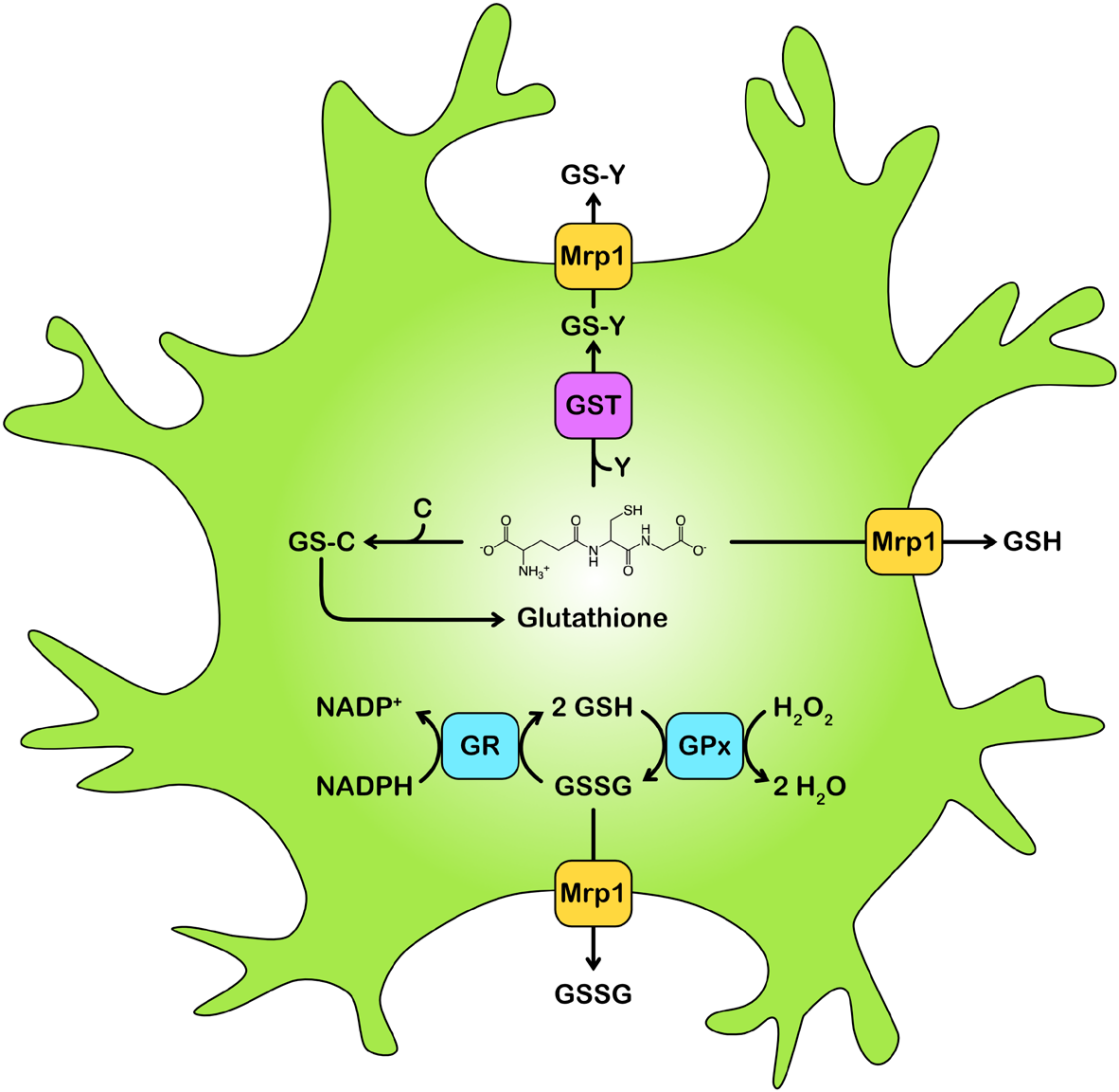
GSH‐dependent processes in astrocytes: Oxidation, regeneration, conjugation, and export. Astrocytic GSH is used for the detoxification of peroxides, electrophiles, and reactive carbonyls, but can also be exported from astrocytes. GSH forms conjugates with electrophilic compounds (Y) in reactions that can be catalyzed by GSH‐S‐transferases (GSTs). GSH is a cofactor of enzymes that detoxify reactive carbonyls (C) such as formaldehyde or methylglyoxal. The GSH used initially to form GSH thiohemiacetals (GS‐C) in such reactions is regenerated in isomerization reactions. The GSH‐dependent reduction of peroxides by glutathione peroxidases (GPx) generates GSSG, which is rapidly reduced back to GSH by glutathione disulfide reductase (GR) that uses NADPH as an electron donor. Alternatively, accumulated GSSG can be exported from astrocytes. The astrocytic export of GSH, GSSG, and GS‐Y is mainly mediated by the multidrug resistance protein 1 (Mrp1).

The intracellular level of GSH is determined by the rates of GSH synthesis and GSH consumption. If GSH synthesis is inhibited by either depletion of the incubation medium of GSH precursors or by the presence of the GCL inhibitor buthionine sulfoximine (BSO), the GSH content of cultured murine astrocytes declines with a half‐time in the hour range (Arend et al. 2024; Devesa et al. 1993; Liddell et al. 2010), demonstrating that astrocytes continuously consume GSH and that this consumption has to be matched by appropriate synthesis of GSH to maintain a constant GSH concentration in the cells. For cultured astrocytes, the processes that are mainly responsible for the consumption of intracellular GSH are—depending on the experimental conditions applied—the oxidation of GSH to GSSG by GPx, the conjugation of GSH with electrophiles, and/or the export of GSH, GSSG, and GSH‐conjugates (Figure 2).

### 3.1 Oxidation of GSH to GSSG

GSH provides the electrons needed for the enzyme‐independent chemical reduction of radicals as well as for the GPx‐catalyzed reduction of peroxides (Figure 2). Both types of reactions oxidize GSH to GSSG that can accumulate transiently in cultured rat astrocytes during treatments that cause oxidative stress (Arend et al. 2024; Dringen and Hamprecht 1997; Watermann and Dringen 2023; Watermann et al. 2025). However, the GSSG generated in astrocytes is rapidly reduced back to GSH by the flavoenzyme glutathione reductase (glutathione disulfide reductase; GR) (Dringen et al. 2015) that uses NADPH as an electron donor (Dringen et al. 2007).

Of the many mammalian isoforms of GPx, especially the cytosolic isoform GPx1 is responsible for the detoxification of hydrogen peroxide and organic hydroperoxides in mouse astrocyte cultures (Liddell, Dringen, et al. 2006; Liddell, Hoepken, et al. 2006). The expression of the isoform GPx4, which is strongly involved in the reduction of lipid peroxides in the context of ferroptosis (Costa et al. 2023; Savaskan et al. 2007; Zhang, Shi, et al. 2024), is strongly upregulated in astrocytes in brain disorders. In addition to GPx, catalase (Dringen and Hamprecht 1997; Liddell et al. 2004, 2010) and peroxiredoxins (Dowell and Johnson 2013; Lu et al. 2019) are expressed in murine astrocytes and contribute to the remarkable ability of these brain cells to efficiently detoxify applied peroxides with half‐times in the minute range (Dringen and Hamprecht 1997; Dringen, Kussmaul, et al. 1999; Dringen et al. 1998a; Kussmaul et al. 1999; Liddell et al. 2004, 2010).

Cultured rat astrocytes have been reported to strongly accumulate GSSG after application of severe oxidative stress, for example, stress induced by the application of a bolus of peroxides (Dringen and Hamprecht 1997; Dringen et al. 1998a; Kussmaul et al. 1999), of a peroxide‐generating system (Hirrlinger et al. 2001) or of compounds that generate radicals due to their autoxidation or metabolism, such as dopamine (Hirrlinger et al. 2002a), menadione (Arend et al. 2024; Ehrke et al. 2021; Raabe et al. 2019; Stapelfeldt et al. 2017; Steinmeier and Dringen 2019) or β‐lapachone (Steinmeier et al. 2020; Watermann and Dringen 2023). For such conditions, the rate of oxidation of GSH to GSSG by GPx is much faster than the rate of reduction of GSSG to GSH by GR (Figure 2). As a consequence, GSSG will accumulate in the cells, and subsequently, also glutathione‐protein mixed disulfides and thiol oxidized proteins will be formed (Aoyama 2021; Ren et al. 2017). However, as soon as the transient oxidative stress is diminished, for example, by cell‐dependent clearance of applied peroxide, GR will efficiently reduce the accumulated GSSG to GSH, thereby reestablishing the initial high ratio of GSH to GSSG. In addition, glutaredoxins and thioredoxins will regenerate the proteins that had been oxidized during the presence of a high intracellular GSSG concentration (Aoyama 2021; Ren et al. 2017).

Astrocytic GR has low micromolar K_M_‐values for NADPH and GSSG and is highly active in astrocytes (Gutterer et al. 1999; Watermann and Dringen 2023). The NADPH needed for GR‐dependent GSSG reduction is regenerated in glucose‐fed cultured astrocytes mainly by the cytosolic pentose‐phosphate pathway (PPP) (Dringen et al. 2007; García‐Nogales et al. 1999; Takahashi 2021; Watermann and Dringen 2023). Accordingly, the moderate GSSG accumulation observed after induction of oxidative stress was found strongly increased after inhibition of GR by carmustine or zinc ions (Bishop et al. 2007) or after inhibition of the PPP by G6PDi‐1 (Watermann et al. 2023). However, the rapid GSSG accumulation reported for such conditions is only transient as GSSG is efficiently exported from cultured murine astrocytes via Mrp1 (Figure 2) (Arend et al. 2024; Hirrlinger et al. 2001, 2002a; Minich et al. 2006; Raabe et al. 2019; Watermann et al. 2025).

### 3.2 Conjugation of GSH to Electrophiles

GSH is a substrate for the detoxification of endogenous and exogenous electrophiles (Figure 2). Accordingly, a large number of different compounds have been reported to lower the GSH content of astrocytes (Dringen et al. 2015). The thiol group of GSH can either directly react chemically with highly reactive compounds, as shown for example for alkylated fumarates (Schmidt and Dringen 2010) or dimethyl itaconate (Watermann et al. 2025). Alternatively, the conjugation reactions can be catalyzed in cells by GSTs (Aloke et al. 2024; Mazari et al. 2023). The GSH adducts formed in such reactions are in most cases less toxic than the unconjugated substrates, and the GSH conjugates can in many cases be efficiently removed from the cells by multidrug resistance protein (Mrp) ‐mediated export (Figure 2) (Dringen et al. 2015). A large variety of different isoforms of GSTs that are divided into several subclasses are known (Aloke et al. 2024; Mazari et al. 2023). Concerning astrocytes and the brain, the available information on the expression and the physiological functions of GSTs and alterations in GST expression and functions in the brain under physiological and pathological conditions has previously been summarized (Dringen et al. 2015; Kumar et al. 2017). More recently, it was reported that GST expression levels are connected with astrocytic activation during inflammation (Matoba et al. 2023).

GSH is also a cofactor of enzymes that detoxify the reactive carbonyls formaldehyde and methylglyoxal (Dringen et al. 2015). For the brain, efficient detoxification of these reactive carbonyls is important as disturbances of the respective detoxification pathways have been connected with neurological disorders and aging (Hambsch 2011; Tulpule and Dringen 2013; Xue et al. 2011). Astrocytes in culture have been shown to efficiently metabolize and thereby detoxify both formaldehyde (Tulpule and Dringen 2013; Tulpule et al. 2013) and methylglyoxal (Bélanger et al. 2011). However, the GSH consumed for the initial reactions that are catalyzed by formaldehyde dehydrogenase and glyoxalase 1 will not cause a loss in GSH content as the GSH used as substrate for the initial reactions is regenerated (Figure 2) during the further reactions (Allaman et al. 2015; Dringen et al. 2015; Tulpule and Dringen 2013).

### 3.3 Export of GSH and GSH Conjugates

Export of GSH (Figure 2) is the main consumer of GSH in unstressed cultured astrocytes. These cultures release around 10% of their GSH per hour (Arend et al. 2013; Dringen et al. 1997). The GSH export from murine astrocytes is primarily mediated by Mrp1 (Hirrlinger et al. 2002b; Minich et al. 2006), which belongs to the ATP binding‐cassette (ABC) protein family of ATP‐driven export pumps (Cole 2014; Huang and Ecker 2023; Wang et al. 2021). Under some conditions, gap junction hemichannels may also contribute to the GSH release from cultured astrocytes (Rana and Dringen 2007; Stridh et al. 2010; Ye et al. 2015). Substantial GSH export is only found for astrocytes, while rates of GSH release from other types of cultured brain cells are much lower (Hirrlinger et al. 2002b; Ye et al. 2015).

In the presence of suitable precursors for GSH synthesis, cultured astrocytes can fully compensate for the export‐mediated GSH loss by GSH *de novo* synthesis, even if Mrp1‐mediated GSH export was further stimulated (Arend et al. 2013). In contrast, inhibition of GSH export in astrocytes can lead to increased GSH levels, as demonstrated by the higher specific GSH content found in Mrp1‐deficient astrocytes compared to wild‐type cultures (Minich et al. 2006).

The velocity of GSH export from cultured murine astrocytes depends strongly on the intracellular concentration of GSH. This export follows apparent Michaelis–Menten kinetics with a K_M_‐value between 25 and 50 mM (Arend et al. 2024; Sagara et al. 1996; Tulpule et al. 2012). Since the cytosolic concentration of GSH in unstressed cultured astrocytes is around 8 mM (Dringen and Hamprecht 1998), any treatment that increases the GSH concentration will also accelerate astrocytic GSH export, as demonstrated by the pre‐incubation of cultured astrocytes with compounds such as ammonium (Murthy et al. 2000), arsenate (Tulpule et al. 2012), arsenite (Sagara et al. 1996), cadmium chloride (Sagara et al. 1996; Tulpule et al. 2012), copper chloride (Scheiber and Dringen 2011; Tulpule et al. 2012), copper oxide nanoparticles (Bulcke and Dringen 2015), nitric oxide (Gegg et al. 2003) or fibroblast growth factor 1 plus tertiary butyl hydroquinone (Vargas et al. 2006). Also, an upregulation of astrocytic Mrp1 expression can accelerate the GSH export as shown for cultured astrocytes that had been treated with the HIV1 envelope glycoprotein gp120 (Ronaldson and Bendayan 2008) or monomeric Aβ‐peptide (Ye et al. 2015). In contrast, treatments that lower astrocytic GSH content, such as inhibition of GSH synthesis by a BSO preincubation or depletion of cystine (Arend et al. 2024; Sagara et al. 1996; Tulpule et al. 2012), lower the rate of GSH export from cultured astrocytes.

GSH export from cultured astrocytes can also be stimulated by exposure to various substances without a preliminary increase in GSH content. These include structurally very diverse substances such as formaldehyde (Tulpule et al. 2012), low concentrations of the Mrp1‐inhibitor MK571 (Hirrlinger et al. 2002b; Minich et al. 2006), antiretroviral protease inhibitors such as ritonavir (Arend et al. 2013, 2024; Brandmann et al. 2012), arsenate and arsenite (Meyer et al. 2013; Tadepalle et al. 2014) as well as octyl itaconate (Watermann et al. 2025). The stimulated astrocytic GSH export induced by these compounds is prevented by inhibition of Mrp1, demonstrating that this transporter mediates the drug‐induced stimulated GSH export, while the basal GSH export is only inhibited by around 60% in the presence of the Mrp1 inhibitor MK571 (Hirrlinger et al. 2002b; Minich et al. 2006). Interestingly, two distinct mechanisms have been reported for the stimulation of Mrp1‐mediated GSH export from cultured astrocytes. Formaldehyde strongly increases the V_max_‐value of the export process by a factor of ten, while the K_M_‐value remains unchanged (Tulpule et al. 2012). This suggests that a formaldehyde treatment initiates recruitment of additional transporter molecules from Mrp1‐containing vesicles into the plasma membrane, as previously reported for bilirubin‐treated astrocytes (Gennuso et al. 2004). In contrast, the presence of the protease inhibitor ritonavir strongly increases the export of GSH from rat astrocyte cultures by lowering the K_M_‐value of Mrp1‐mediated GSH export (Arend et al. 2024).

In addition to GSH export, cultured astrocytes are able to efficiently export GSSG and GSH‐conjugates via Mrp1 (Figure 2) under conditions that induce oxidative stress (Arend et al. 2024; Hirrlinger et al. 2001; Minich et al. 2006; Raabe et al. 2019;) and after the application of given electrophiles (Arend et al. 2024; Dringen et al. 2015; Raabe et al. 2019; Waak and Dringen 2006), respectively. However, the kinetic parameters involved in the export of GSH, GSSG, and GSH‐conjugates from astrocytes differ strongly from each other. While the K_M_‐value for GSH export from astrocytes is in the higher millimolar range (Arend et al. 2024; Tulpule et al. 2012), K_M_‐values for the Mrp1‐mediated export of GSSG and the GSH‐conjugate glutathione‐bimane (GS‐B) are in the micromolar and nanomolar range (Homma et al. 1999; Leier et al. 1996), respectively. Although GSH, GSSG, and GS‐B are exported by Mrp1, as demonstrated by the strong impairment of the respective export process by inhibition or knockout of Mrp1 (Hirrlinger et al. 2002b; Minich et al. 2006), a ritonavir‐induced export stimulation was only found for the export of GSH (Arend et al. 2013, 2024; Brandmann et al. 2014), while ritonavir blocks the Mrp1‐mediated GSSG and GS‐B export from cultured rat astrocytes (Arend et al. 2024).

## 4 Functions of Astrocytic GSH for Neurons and the Brain

Astrocytes have a high GSH content which enables these cells to efficiently perform GSH‐dependent processes, such as the reduction of potential harmful peroxides and removal of xenobiotics (Figure 2) (Dringen et al. 2015). Accordingly, a lowered GSH content slows down the clearance of peroxides (Dringen and Hamprecht 1997; Dringen et al. 1998b; Kussmaul et al. 1999; Liddell, Dringen, et al. 2006; Liddell, Hoepken, et al. 2006) and compromises the resistance of cultured murine astrocytes toward the damage induced by oxidative stress, xenobiotics or toxic metals (Table 1). Thus, a high GSH content is beneficial for astrocytes and will thereby help to maintain the many important physiological functions that astrocytes have in the brain.

**TABLE 1:** Compromised resistance of GSH‐depleted cultured astrocytes against adverse treatments.

Adverse consequence of treatment	Species	GSH depletion by exposure to	Methods used for analysis	References
Peroxynitrite‐induced inactivation of mitochondrial respiratory chain complexes	Rat	BSO	Enzyme assays	Barker et al. (1996)
Zink‐induced cytotoxicity	Mouse	BSO	LDH	Kim et al. (2003)
Cytotoxicity induced by polybrominated diphenyl ether mixture DE71	Mouse	BSO	MTT	Giordano et al. (2008)
Ischemia‐induced apoptotic cell death	Rat	BSO	MTTApoptotic nuclei	Gabryel and Małecki (2006)
Methylmercury‐induced cytotoxicity	Mouse	Diethyl maleate	MTT ROS staining	Kaur et al. (2006)
Methyl mercury‐induced ROS‐production	Rat	BSO	ROS staining	Shanker et al. (2005)
Cadmium‐induced cytotoxicity	Mouse	BSO	LDH	Im et al. (2006)
Peroxide‐induced iron cytotoxicity	Mouse	BSO	LDH	Liddell, Dringen, et al. (2006)
	Mouse	BSO	LDH	Liddell, Hoepken, et al. (2006)
Peroxide‐induced iron cytotoxicity	Rat	BSO	LDH	Liddell et al. (2004)

Abbreviations: BSO, buthionine sulfoximine; LDH, lactate dehydrogenase release assay; MTT; MTT reduction assay; ROS, reactive oxygen species.

Cultured astrocytes from murine brain have frequently been reported to contain higher specific GSH contents than cultured neurons (Dringen, Kussmaul, et al. 1999; Dringen et al. 2005; Vázquez‐Meza et al. 2023). The likely reason for this observation is a different preference of astrocytes and neurons to use extracellular amino acids as precursors for the intracellular substrates needed for GSH synthesis. A combination of extracellular glutamate and cystine enables cultured rat astrocytes to most efficiently synthesize GSH, while cultured rat neurons prefer glutamine and cysteine as extracellular precursors for GSH synthesis (Kranich et al. 1996).

A high astrocytic GSH content of astrocytes is essential to provide neighboring neurons with amino acid precursors for neuronal GSH synthesis. Several groups have shown that in co‐cultures, the presence of astrocytes increases the GSH content in the cocultured neurons (Bolaños et al. 1996; Diaz‐Hernandez et al. 2005; Dringen, Pfeiffer, et al. 1999; Gegg et al. 2005; Pizzurro et al. 2014; Rathinam et al. 2012), demonstrating that the presence of astrocytes has a stimulating positive effect on the GSH content of neurons. The limiting substrate for the synthesis of GSH in neurons is the amino acid cysteine. In contrast to astrocytes, neurons appear not to take up and use substantial amounts of cystine for GSH synthesis and rely therefore on the availability of cysteine as an extracellular precursor for the cysteine moiety of GSH (Kranich et al. 1996).

The cysteine supply by astrocytes is initiated by the export of GSH from astrocytes (Dringen, Pfeiffer, et al. 1999; Wang and Cynader 2000) which is mainly mediated by the ATP‐driven Mrp1 (Hirrlinger et al. 2002b; Minich et al. 2006). The exported GSH becomes substrate of the astrocytic ectoenzyme γGT which liberates the dipeptide CysGly. The supply of cysteine as neuronal GSH precursor by astrocytes (Figure 1) is strongly inhibited by the presence of the γGT inhibitor acivicin (Dringen, Pfeiffer, et al. 1999), demonstrating that γGT plays a central role in the supply of cysteine to neurons. CysGly appears not to be directly taken up by neurons but is rather hydrolyzed extracellularly before the liberated amino acids cysteine and glycine are taken up into neurons (Figure 1). Responsible for the hydrolysis of CysGly is the neuronal aminopeptidase N, as demonstrated by the ability of an inactivating specific antibody against aminopeptidase N to prevent the utilization of CysGly for neuronal GSH synthesis (Dringen et al. 2001). The amino acids liberated by the hydrolysis, cysteine and glycine, are efficiently taken up by sodium‐dependent transporters into neurons (Aoyama and Nakaki 2013; Zafra and Giménez 2008) and can be used as substrates for neuronal GSH synthesis (Dringen, Pfeiffer, et al. 1999; Kranich et al. 1996).

Exported GSH has also been shown to reduce extracellular cystine, thereby liberating cysteine (Wang and Cynader 2000) and this process has been discussed to contribute to the supply of cysteine for neuronal GSH synthesis (Pérez‐Sala and Pajares 2023; Wang and Cynader 2000). Furthermore, transport of GSH and/or GSH precursors via astrocyte‐derived extracellular vesicles to neurons has been proposed as a process to supply neurons with GSH precursors (Pérez‐Sala and Pajares 2023). However, both the chemical reduction of extracellular cystine by GSH released from astrocytes as well as transfer via extracellular vesicles should not be affected by inhibitors of the astrocytic γGT or neuronal aminopeptidase N, which were shown to be involved in the supply of cysteine to neurons (Dringen, Pfeiffer, et al. 1999) and in the use of CysGly by neurons (Dringen et al. 2001). It remains to be elucidated to which extent and under which conditions the ectoenzyme‐dependent (Figure 1) and ‐independent pathways may contribute to the supply of cysteine for the GSH synthesis in neurons under physiological or pathological conditions.

The supply of GSH precursors from astrocytes to neurons does not only increase the GSH content in neurons but also improves the resistance of neurons to various adverse treatments, including induction of oxidative stress and application of toxins (Table 2), demonstrating the importance of the astrocytic cysteine supply for neuronal GSH synthesis. Interestingly, improved supply of GSH precursors from astrocytes to neurons and a thereby better resistance of neurons has been reported after β‐adrenergic stimulation (Yoshioka et al. 2021), by exposure to interleukin 1β (Chowdhury et al. 2018) or after exposure to ethanol (Rathinam et al. 2006). An important regulator of the cysteine supply from astrocytes to neurons could also be the prion protein (PrP). PrP‐deficient cerebellar neurons show impaired survival after exposure to hypoxia or treatments with glutamate or H_2_O_2_, while PrP‐deficient astrocytes have enhanced GSH contents and show accelerated GSH export and reduced γGT activity (Guitart et al. 2015).

**TABLE 2:** Protection of neurons against adverse treatments by the GSH metabolism of cocultured astrocytes.

Adverse consequence of a modified GSH metabolism of astrocytes on cocultured neurons	Species	Modulated astrocytic GSH metabolism	Methods used to detect neuronal impairment	References
P75^NTR^‐dependent motor neuron toxicity	Rat	Inhibited GSH release from astrocytes	Counting neurons	Vargas et al. (2006)
Organophosphorous insecticides‐induced impaired neurite outgrowth	Rat	Lowered GSH content by BSO preincubation	Measurement of neurite length	Pizzurro et al. (2014)
Glutamate‐induced neurotoxicity	Rat	Lowered GSH content by BSO preincubation	Counting neurons	Shih et al. (2003)
Neurotoxicity induced by polybrominated diphenyl ether	Mouse	Lowered GSH content by GCSm deficiency	MTT	Giordano et al. (2009)
Hydrogen peroxide‐induced neurotoxicity	Mouse	Inhibited GSH release from astrocytes	Counting neurons	Yoshioka et al. (2021)
Ethanol‐induced neuronal ROS formation	Rat	Inhibition of astrocytic γGT	ROS staining of neurons	Rathinam et al. (2006)
Neurotoxicity induced by rotenone and paraquat	Rat	Inhibition of astrocytic γGT	Counting of Annexin‐positive cells	Rathinam et al. (2012)

Abbreviations: γGT, γ‐glutamyl transpeptidase; BSO, buthionine sulfoximine; MTT, MTT reduction assay; ROS, reactive oxygen species.

The supply of GSH precursors from astrocyte appears not to be exclusive for neurons as receiving cell type. A similar interaction in GSH metabolism by cysteine supply via GSH export appears to take place between astrocytes and endothelial cells, and this cysteine shuttle has been reported to help to maintain endothelial barrier stability and integrity (Huang et al. 2020). Further studies are now requested to test whether astrocyte‐derived GSH may also help to maintain GSH levels in other types of brain cells, including oligodendrocytes and microglial cells.

## 5 Future Perspectives

Astrocytes have been shown to play a central role in the GSH metabolism of the brain and provide neighboring brain cells with cysteine as an essential substrate for GSH synthesis. Most of the processes involved in the shuttling of GSH substrates from astrocytes to other types of brain cells have been studied on cultured murine brain cells. So far, little information is available on the metabolic interactions of astrocytes and neurons in the human brain (Ciani et al. 2024), which is especially the case for GSH metabolism. Thus, studies are now required to confirm that human astrocytes also provide GSH precursors to neighboring cells. In addition, it should be confirmed that cysteine supply by astrocytes to neurons plays an important role in the intact brain. For example, targeting specifically the astrocytic processes involved in the cysteine supply by generating mouse models with astrocyte‐specific knockdowns of the Mrp1‐mediated GSH export or the extracellular processing of GSH by γGT may provide confirmation that astrocytes also provide other brain cell types with GSH precursors in vivo.

Also, the signaling processes that are involved in the regulation of the GSH metabolism in the brain and in the supply of cysteine from astrocytes to neurons remain an interesting topic to study. An induction of the expression of transporters that mediate the uptake of GSH precursors and/or of the astrocytic enzymes that are involved in the cysteine supply to neurons may improve the supply of the GSH precursor cysteine from astrocytes to neighboring brain cells and thereby help to prevent neurotoxicity in pathological conditions.

Extracellular GSH concentrations in the brain are very low (Hilgier et al. 2010; Orwar et al. 1994), most likely due to the rapid metabolism of released GSH by γGT. In addition to GSH‐derived CysGly, which can serve as a cysteine precursor for neurons (Figure 1), the γGT produces γ‐glutamyl peptides from extracellular GSH (Orwar et al. 1994). Both GSH and γ‐glutamyl peptides have been discussed as extracellular neuromodulators of glutamate receptors (Janáky et al. 1999). More recently, GSH and γ‐glutamyl peptides have also been connected to taurine release from brain slices (Janáky et al. 2008) and gained considerable interest as modulators of family C‐G‐protein coupled receptors (Wang et al. 2006) and calcium‐sensing receptors (Ikeda and Fujii 2023). Further studies are now required to elucidate the potential of astrocyte‐derived GSH as well as of γ‐glutamyl peptides generated by astrocytic γGT as extracellular modulators of receptor‐mediated signaling in the brain.

The recent progress in developing nanosensors for the visualization of GSH in cells (Bagherpour and Pérez‐García 2024; Waris et al. 2023) makes it quite likely that such tools will be available soon to monitor in real time the alterations in intracellular GSH concentrations and metabolic cooperations in the GSH metabolism between different types of brain cells in cell cultures but also in more complex settings in vitro and in vivo. Such studies may also allow testing whether the astrocytic supply of GSH precursors to other brain cell types is a general feature of all astrocytes or whether the known metabolic heterogeneity of astrocytes (Calì et al. 2024; Ciani et al. 2024; Hasel et al. 2023; Wolff et al. 2025) has also to be considered for the GSH metabolism of the brain.

Oxidative stress and alterations in the GSH metabolism of the brain have been connected with many neurological and neurodegenerative diseases as recently summarized (Asanuma and Miyazaki 2021; Bell et al. 2024; Bjørklund et al. 2021, 2020; Bottino et al. 2021; Iskusnykh et al. 2022; Kim 2021; Murray et al. 2024). For some diseases and some brain regions, a loss in the overall content of GSH has been found by magnetic resonance spectroscopy (MRS) (Bottino et al. 2021; Rae and Williams 2017). However, contradictory results reported by different groups, which may arise in part due to methodological differences, make an evaluation of the literature on alterations in brain GSH levels in human diseases difficult (Bottino et al. 2021; Detcheverry et al. 2023; Rae and Williams 2017). Further research on the methods used to determine GSH levels in the living human brain as well as a better definition of standard procedures for analyzing GSH contents by MRS in the brain are warranted to obtain a more consistent picture on alterations in the GSH levels in the human brain in health and disease. Currently, it is unclear whether and to what extent a disturbed astrocytic GSH metabolism is involved in the reported alterations in brain GSH levels in human neurological diseases. Further studies are also required to elucidate whether an increased GSH level in astrocytes in the brain could elevate the overall GSH content in the brain and whether such an intervention would affect the symptoms or modulate the progression of human neurological diseases.

## Author Contributions

**Ralf Dringen:** conceptualization, software, writing – original draft, writing – review and editing. **Christian Arend:** software, visualization, writing – original draft, writing – review and editing.

## Conflicts of Interest

*Journal of Neurochemistry* (JNC) is owned by the International Society for Neurochemistry (ISN). R.D. was Secretary (2015–2019) and President (2019–2021) of ISN. He is currently an ISN Council member, ISN Historian, and ISN Archivist. Otherwise, the authors have no potential conflict to declare.

## Data Availability

The authors have nothing to report.
